# Type I interferon-dependent gene MxA in perinatal HIV-infected patients under antiretroviral therapy as marker for therapy failure and blood plasmacytoid dendritic cells depletion

**DOI:** 10.1186/1479-5876-6-49

**Published:** 2008-09-09

**Authors:** Raffaele Badolato, Claudia Ghidini, Fabio Facchetti, Federico Serana, Alessandra Sottini, Marco Chiarini, Elena Spinelli, Silvia Lonardi, Alessandro Plebani, Luigi Caimi, Luisa Imberti

**Affiliations:** 1 Istituto di Medicina Molecolare "Angelo Nocivelli", Department of Pediatrics, University of Brescia, Brescia 25123, Italy; 2 Terzo Laboratorio degli Spedali Civili, Department of Diagnostics, Spedali Civili di Brescia, 25123 Brescia, Italy; 3 Department of Pathology, University of Brescia, 25123 Brescia, Italy

## Abstract

**Background:**

To determine the role of interferon-alpha in controlling HIV infection we phenotypically and functionally analyzed circulating plasmacytoid dendritic cells (pDC), which are known to be the highest interferon-alpha producing cells, in 33 perinatally infected HIV^+ ^patients undergoing standard antiretroviral therapy.

**Methods:**

Circulating pDC were identified by flow cytometry using anti-BDCA-2 monoclonal antibody and by measuring BDCA-2 mRNA by real-time PCR, while tissue-resident pDC were identified by immunohistochemistry. mRNA for interferon-alpha and MxA, a gene that is specifically induced by interferon-alpha, was quantified in peripheral blood cells by real-time PCR, while serum interferon-alpha protein was measured by ELISA.

**Results:**

While median values of pDC, both in terms of percentage and absolute number, were not statistically different from age-matched controls, interferon-alpha mRNA was increased in HIV-infected patients. However, in a group of patients with long disease duration, having a low number of both pDC and CD4^+ ^lymphocytes and a significant increase of serum interferon-alpha, MxA mRNA was produced at high level and its expression directly correlated with HIV RNA copy numbers. Furthermore in patients displaying a low CD4^+ ^blood cell count, a severe depletion of pDC in the tonsils could be documented.

**Conclusion:**

HIV replication unresponsive to antiretroviral treatment in perinatal-infected patients with advanced disease and pDC depletion may lead to interferon-alpha expression and subsequent induction of MxA mRNA. Thus, the latter measurement may represent a valuable marker to monitor the clinical response to therapy in HIV patients.

## Background

Over the last 10 years the use of antiretroviral therapy (ART) in HIV-infected children has resulted in noticeable benefit as indicated by increased survival [1,2]. However, poor adherence to prescriptions and the high rates of virus replication that are characteristic of perinatal infection [3] often lead to higher virological set points in children compared to adults and lower rates of attainment of undetectable viral loads. Therefore, the identification of immunological correlates of immune reconstitution and early predictors of antiretroviral failure in HIV-treated children are needed. Concomitantly with loss of CD4^+ ^cells, children with HIV infection display a profound impairment of the innate branch of immune system as indicated by the progressive decrease of circulating dendritic cells (DC). DC are a heterogeneous population of antigen-presenting cells that are required to draw the first line of host defense against viral infections [4-6]. Two subsets of DC were originally identified in peripheral blood on the basis of the β2-integrin expression (CD11c marker): the CD11c^+ ^myeloid DC (mDC) and the CD11c- plasmacytoid DC (pDC). DC phenotype is defined on the basis of the two monoclonal antibodies (mAb) BDCA-1 and BDCA-2, which identify blood mDC and pDC, respectively [7,8]. But, BDCA-1 is also expressed on monocytes and is not sufficient by itself to define dendritic cells [8].

Although blood DC represent less than 1% of total peripheral blood mononuclear cells (PBMC), they exert a relevant protective role against invading pathogens by producing IL-12 and interpheron-alpha (IFN-α) and by inducing T-cell immunity via presentation of pathogen-specific antigens on their cell surface [9-13]. IFN-α has been shown to decrease HIV replication by induction of IFN-stimulated genes. One such gene is Myxovirus resistance 1, which encodes for the Myxovirus resistance protein A (MxA), a protein capable of inhibiting the replication of several viruses, including HIV [14,15]. However, it is controversial whether the increase of IFN-α secretion, which is observed during disease progression, contributes to HIV pathogenesis. It has been described that high levels of IFN-α might exert deleterious effects on the immune cells by inducing depletion of uninfected CD4^+ ^lymphocytes [16-18]. Like CD4^+ ^cells, DC express the receptor machinery necessary for HIV entry, and therefore are vulnerable to the detrimental effects of HIV infection and are functionally impaired in HIV-1-infected patients [19-24]. Furthermore, pDC loss is believed to be a predictor of disease progression, of increased risk of opportunistic infections and of Kaposi sarcoma development [19,20,23,24].

It is known that immune reconstitution after ART is mostly due to newly released lymphocytes from the thymus [25]. However, at the same time, antiretroviral regimens influence DC pool in both adults and children by completely restoring mDC and only partially recovering the frequency and function of pDC [21,26].

In this study we evaluated pDC and IFN-α involvement in a cohort of 33 perinatally HIV-infected patients undergoing ART at various stages of immune-reconstitution, as determined by analysis of their viral load and CD4^+ ^T-cell counts.

## Patients and methods

### Patients and controls

Informed consent was obtained, in accordance with institutional review board guidelines for the protection of human subjects, from parents or legal guardians of 33 subjects with perinatal HIV infection (age: from 2 up to 19 years), and from 15 age-matched healthy controls (mean age: 11.8 years, range: 2–19 years). Patients with acute infectious diseases or opportunistic infections at the time of the analysis were excluded from the study.

### Circulating pDC and serum IFN-α levels determination

The identification of pDC was performed using Blood Dendritic Cell enumeration Kit (Miltenyi Biotec GmbH, Bergish Gladbach, Germany) as previously described [27]. Blood samples were stained simultaneously with anti-BDCA-2-FITC mAb to identify pDC, anti-CD19-PE-Cy5 and anti-CD14-PE-Cy5 mAbs to exclude B cells and monocytes, and with a fluorescent cell-impermeant dye (Dead cells Discriminator), which binds covalently and irreversibly to nucleic acids of dead cells. After cells staining, erythrocytes were lysed with ammonium chloride buffer (Red Blood Cell Lysis), and cells were washed and fixed using formaldehyde (Fix Solution). Finally, cells were analyzed by 3-color flow cytometry using FACScan (Becton Dickinson, San Jose, CA, USA).

The level of IFN-α in the serum was measured by Sandwich ELISA using an IFN-α kit (PBL Biomedical Laboratories, Piscataway, NJ, USA) and following the manufacturer's instructions.

### IFN-α, BDCA-2, MxA and TRECs quantification by real-time PCR

Blood samples (2.5 ml) from patients and controls were drawn into PAXgene tubes (preAnalytiX GmbH, Hombrechtikon, CH), which contain an additive that stabilizes gene transcription profile by reducing *in vitro *RNA degradation and minimizing gene induction. RNA was prepared using PAXgene Blood RNA kit following manufacturer's instructions (preAnalytiX). To remove contaminating DNA, samples were treated with DNase (Qiagen Inc. Valencia, CA, USA). Five hundred ng of total RNA were reverse transcribed with random hexamers in a final volume of 20 μl using the TaqMan RT kit (Applied Biosystems, Foster City, CA, USA). Real-time PCR analysis was performed with the GeneAmp 5700 Sequence Detection System (Applied Biosystems). For IFN-α and BDCA-2 mRNA measure a pre-developed TaqMan assay, which included the appropriate primer/probe combination, was purchased from Applied Biosystems and the test was performed following manufacturer's instructions. For MxA mRNA quantification we used primers and probes described by Pachner et al. [28]; the experiments were performed after standardization of the assay in terms of precision, accuracy and reproducibility [29]. All PCR reactions were set up in 96-well optical reaction plates (ABgene, Epsom, UK) in a final volume of 25 μl with 12.5 μl of double concentrated TaqMan universal PCR Master mix (Applied Biosystems), 3 μl of cDNA, 1.25 μl of primer/probe mix for IFN-α and BDCA-2 and 600 nM of primers and 200 nM of probe for MxA. Probe and primer concentration for the housekeeping gene, Glyceraldehyde-3-phosphate dehydrogenase (GAPDH), were respectively 400 and 200 nM. PCR amplification started with a first step at 50°C for 2 min., followed by an initial heating at 95°C for 10 min.; samples were then subjected to 45 cycles of denaturation at 95°C for 15 sec and annealing at 60°C for 1 min. Each sample was run in duplicate.

The relative quantification of BDCA-2, IFN-α and MxA mRNA expression was calculated using the Comparative cycle threshold (Ct) method according to the following formula: Normalization Ratio (NR) = 2^-ΔΔCtCt^. First, for each sample, ΔCt value was calculated as the Ct of the target gene minus the Ct of GAPDH and then the ΔΔCt value was obtained as the difference between the ΔCt of the sample and the ΔCt of the calibrator. According to the formula, the normalization ratio of the calibrator in each run is 1. As calibrator in each sample run, we utilized the same RNA extracted from a single healthy control, and stored at -80°C.

The quantification of the signal-joint T-cell receptor excision circles (TRECs) was performed on PBMC separated by Ficoll-Hypaque density gradient centrifugation using the standard curve method described by Pirovano et al. [30].

### Immunohistochemistry

Lymph node and tonsil specimens from 8 patients with HIV infection and from 2 age-matched controls were analyzed. Each specimen was fixed in buffered formalin and embedded in paraffin. Sections were stained overnight with a dilution 1:50 of mouse anti-CD123 mAb (clone 7G3, IgG2a, Becton Dickinson PharMingen Biosciences Europe, Erembodegem-AALST, Belgium), after inhibition of endogenous peroxidase activity with 0.3% H_2_O_2 _in methanol for 20 min and heat-induced epitope retrieval in Tris-EDTA buffer (pH 9.0). Reaction was developed with the polymer peroxidase conjugated technique (ChemMate, Dako, Glostrup, DK) and diaminobenzydine as chromogen. Sections were counterstained with haematoxylin. Cell counting was performed using an Olympus BX60 microscope equipped with the Olympus DP-70 digital camera and the Olympus Soft Imaging System Cell-F 2.5; two medium-power fields (corresponding to 0.3 mm^2^), selected on the basis of highest content of positive cells, were evaluated and values were expressed as mean per one mm^2^.

### Statistical analysis

Since real-time PCR data did not follow a Gaussian distribution, results were expressed as median and range and analyzed using nonparametric statistical tests. Differences between values from patients and controls were examined by Mann-Whitney test, while comparisons between more than 2 groups of results were assessed by Kruskal-Wallis ANOVA. In case of significance, Dunn's multiple columns post-test was applied to compare the sub-groups of treatment. Moreover, Spearman's correlation test was used to find an association between variables. Two-tailed P values < 0.05 were considered to be statistically significant.

## Results

### Clinical and immune features of perinatally HIV- infected patients

We evaluated pDC number and function in a population of 33 perinatally HIV-infected patients, 17 of them were female and 16 male. CD4^+ ^T lymphocytes ranged from 18.6% to 43.4% (median of 31.6%; Table 1). In particular, 26 of 33 subjects showed an adequate immune reconstitution (Category 1, CD4^+^% ≥ 25%), while the remaining patients had a low number of CD4^+ ^and a high viral load. They all were under ART at the time of the analysis according to PENTA guidelines for treatment of HIV infection in children [31], but the adopted treatment regimen varied from subject to subject depending on the pattern of genotypic resistance and on the adherence to the therapy [32]. Twenty-four patients were subjected to ART regimen containing at least one protease inhibitor, 7 patients were treated with a non-nucleoside reverse transcriptase inhibitor, and the remaining 2 patients with two nucleoside reverse transcriptase inhibitors because of their poor adherence to other regimens. Despite the adoption of ART, complete suppression of viral load (less than 50 copies/ml) was achieved only in 18 out of 33 patients (Table 1). This unsatisfactory result was probably the consequence of several contributing reasons, including the common observance of genotypic resistance in children failing the treatment, the long length of the disease in older patients, the slow reduction of viral load following the introduction of ART in children, and possibly, the difficulty in obtaining long-term adherence to therapy in adolescents [2,3].

**Table 1:** Clinical and immunological features of HIV-infected children

**Patient ID**	**Age (years)**	**Sex**	**CD4^+ ^(%)**	**HIV RNA (copies/ml)**	**CDC**
1	16	F	18.6	18527	B 3
2	14	M	18.8	12461	B 2
3	19	F	20	50	B 3
4	19	F	20	4506	B 2
5	17	F	21.3	78	C2
6	8	M	21.9	4366	B 2
7	18	M	23.9	65	A 3
8	16	M	25.2	50	B 3
9	4	F	25.6	166	B 1
10	16	F	28.9	50	B 3
11	16	M	29.1	14627	A 2
12	14	F	30	50	B 3
13	11	F	30.2	50	C 2
14	8	M	30.5	50	A 2
15	8	M	30.7	50	A 2
16	4	M	30.8	2214	C 2
17	12	M	31.6	50	C 3
18	17	M	31.9	50	B 3
19^a^	12	F	32	50	C 2
20	11	F	32.6	50	A 2
21	2	M	33	2154	A1
22	13	F	33.3	50	A 2
23	4	M	33.5	6984	N 1
24	6	M	33.9	50	B 2
25^a^	16	F	34.5	50	A 2
26	10	M	34.6	77	A 2
27	11	F	38.2	516	B 1
28	11	M	39.3	50	C 1
29	9	F	40.9	50	B 1
30	9	F	41	5674	B 1
31	14	M	42.2	50	C 3
32^a^	9	F	43.4	50	B 2
33^a^	15	F	43.4	21422	B 2

^a^Children with HCV co-infection

### pDC in HIV-infected perinatally HIV-infected patients

**Figure 1:**
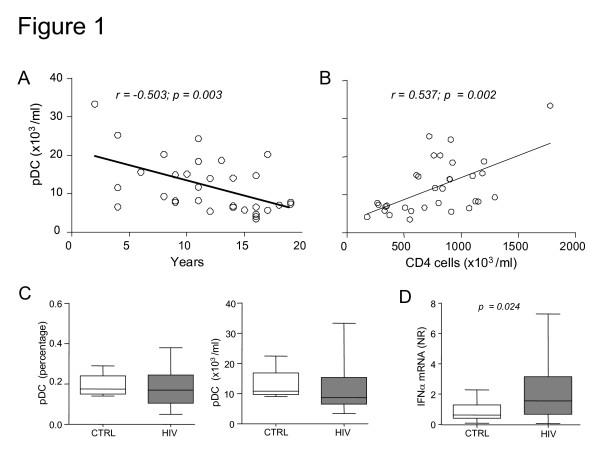
**(A) Correlation of pDC with age and (B) with CD4^+ ^cell number in ART-treated HIV^+ ^patients. (C)** Percentage and number of pDC in ART-treated HIV^+ ^children and in age-matched controls (CTRL). **(D)** Values of IFN-α mRNA, obtained by real-time PCR and expressed as normalization ratio (NR), in HIV^+ ^children and CTRL.

We evaluated both the proportion and the absolute number of pDC in HIV-infected patients by using an assay that selectively identified the BDCA-2^+^CD14^-^CD19^- ^cell subset. As observed in healthy controls [33], there is an inverse correlation (r = -0.503, p = 0.003) of pDC values with age (Figure 1A) and a direct correlation (r = 0.537; p = 0.002) with the absolute cell number of CD4^+ ^T lymphocytes (Figure 1B). The progressive decline of pDC (values ranging from 33,300 cells/μl to 3,402 cells/μl) can reflect the "natural" decline in pDC counts during ageing [33] and is related to the reduction of CD4^+ ^cell number. This is in agreement with data from Azzoni et al. [34], demonstrating a greater depletion of pDC in HIV^+ ^children with a clinical history of decreasing CD4^+ ^cell percentage. Median values of pDC, both in terms of percentage and absolute number, are not statistically different from those measured in age-matched controls, although pDC number of HIV^+ ^patients receiving ART is heterogeneous and varies from extremely low blood counts up to levels observed in controls (Figure 1C).

Since BDCA-2 was shown to be modulated following pDC activation [7], we have evaluated the amount of BDCA-2 mRNA by real-time PCR. Comparable values were found in patients and healthy controls (median value of: 1.13 in HIV^+ ^patients vs. 0.93 in healthy children, p = NS), thus confirming the data obtained by cytofluorimetry. We did not assess pDC counts before treatment in our patients, but based on previous observations we speculate that pDC counts, possibly lower before treatment, might be increased after starting the ART.

### IFN-α and MxA mRNA induction during HIV infection

**Figure 2:**
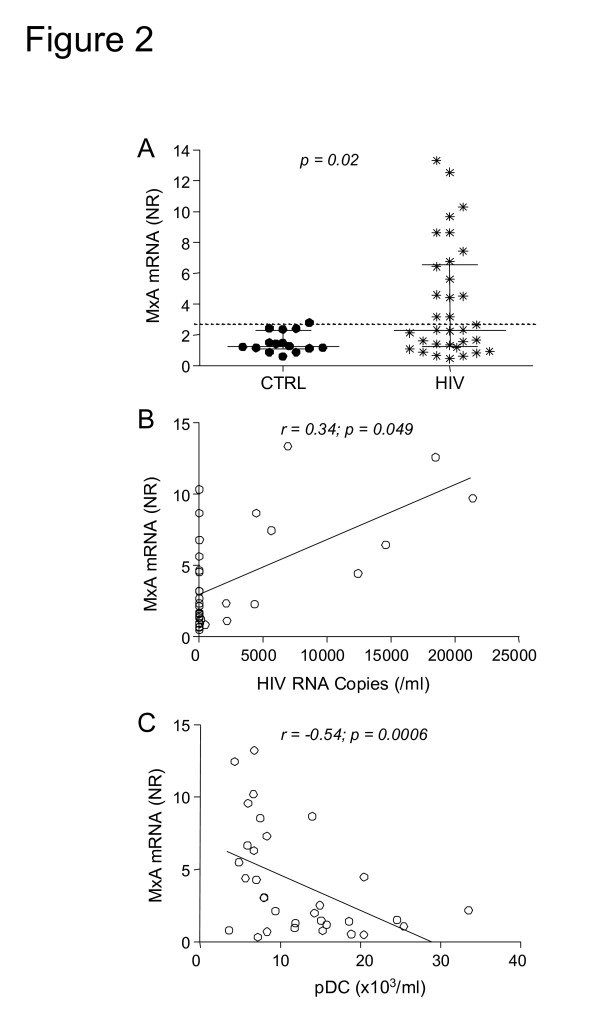
**(A) MxA mRNA levels in ART-treated HIV^+ ^children and controls (CTRL).** Correlation between MxA mRNA levels and HIV viral load **(B)** and pDC number **(C)**. NR: normalization ratio.

**Figure 3:**
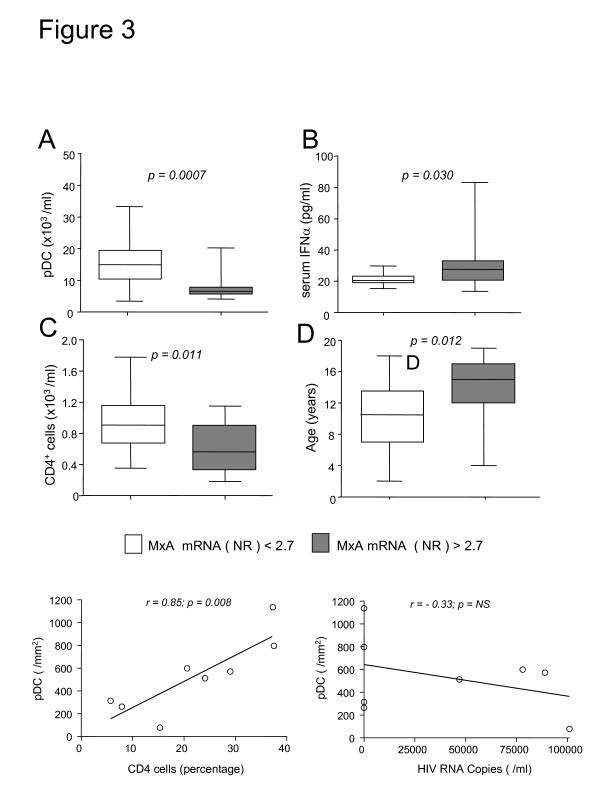
**Immunological parameters of 2 groups of ART-treated HIV^+ ^patients divided according the production of MxA mRNA in MxA-non induced (cut-off value NR < 2.7) and MxA-induced (NR > 2.7) patients. (A)** Number of pDC, **(B)** level of serum IFN-α, **(C)** number of CD4^+ ^lymphocytes, and **(D)** age of the patients. NR: normalization ratio.

Since pDC are specialized cells that produce large amounts of IFN-α in response to viral infections, we sought to determine whether HIV infection induces the expression of this cytokine. We observed a significant increase of IFN-α mRNA in PBMC in HIV-infected patients as compared to healthy control subjects (Figure 1D), suggesting that the chronic infection induces IFN-α production. Next, we analyzed the expression of MxA mRNA, which is selectively induced after type-I IFN receptor binding and is a marker for cell responsiveness to IFN-α [14,35]. We found that MxA mRNA levels were significantly increased in HIV-infected patients with abnormal HIV viral load RNA. Indeed, we observed a direct correlation between MxA expression and HIV RNA copies in HIV infected patients (Figure 2B), suggesting that a high number of viral copies may directly influence the induction of MxA mRNA. Due to the fact that pDC are one of the principal producers of IFN-α [9] and that MxA is a marker of type I IFN bioactivity [29,35,36], one might predict that patients with the largest number of circulating pDC might express more MxA as compared to age-matched controls (Figure 2A). On the contrary, we have observed that higher levels of MxA mRNA are found in those patients with lower number of circulating pDC, as indicated by the strong negative correlation between these two parameters (Figure 2C). In particular, we observed that 15 patients displayed MxA mRNA levels above the cut-off, established as the 99^th ^percentile of the distribution of MxA in healthy control subjects (NR = 2.7, shown as dotted line in the figure), while the remaining presented normal MxA mRNA expression. Surprisingly, patients with MxA mRNA levels above the cut-off are those with lower numbers of pDC (Figure 3A) and significantly higher serum levels of IFN-α (Figure 3B). It is of note that the median level of this cytokine is low in both groups, at levels observed in control children, but it has been demonstrated that circulating IFN-α is elevated in the sera just at the moment of HIV antigen appearance [37]. Moreover, we have also observed that children with low MxA mRNA (<2.7) below the cut-off show an increased number of CD4^+ ^lymphocyte (Figure 3C) and that children with increased MxA mRNA (>2.7) are of older age (Figure 3D). Since MxA levels do not vary among ages [29,36], we speculate that increased levels of MxA mRNA are associated with the length of HIV infection. Finally, since pDC are present within the thymus [15] and immune-reconstitution is related to thymic function [38], we utilized real-time PCR to determine the levels of DNA episomes created in the thymus during T-cell receptor rearrangement process, known as TRECS. We observed that TREC values were comparable in HIV-infected patients and age-matched controls (63,345 TRECs/1 × 10^6 ^cells, range: 39,470–173,194 vs. 54,560 TRECs/1 × 10^6^, range: 7,279–299,500), without any detectable difference between patients with normal or increased MxA mRNA values (median: 65,925 TRECs/1 × 10^6 ^cells, range: 15,259–155,003 vs. 51,125 TRECs/1 × 10^6 ^cells, range: 17,586–128,900). Similarly, there was no significant correlation between TRECs and pDC cell number (data not shown).

### pDC in lymphoid tissues of HIV infected children

**Figure 4:**
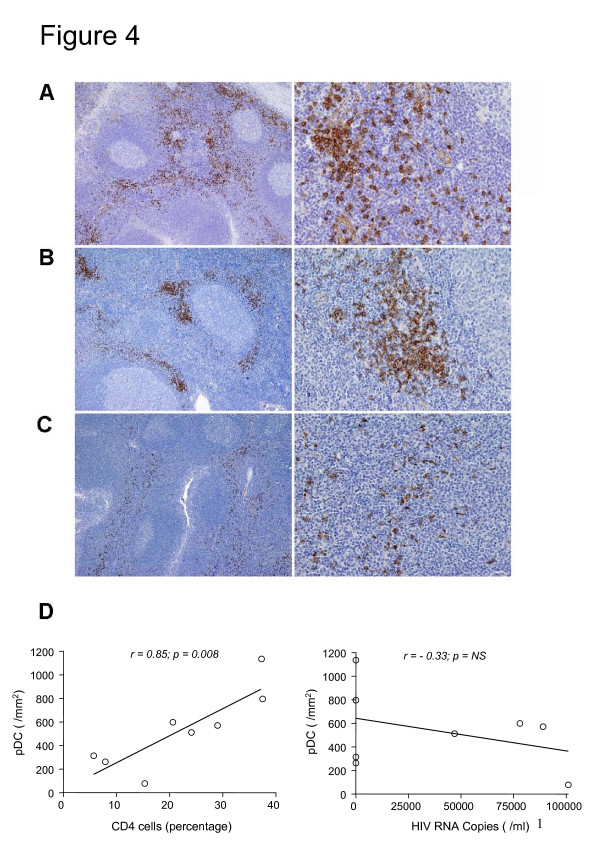
**Analysis of tissue pDC.** pDC counting was performed using an Olympus BX60 microscope equipped with the Olympus DP-70 digital camera and the Olympus Soft Imaging System Cell-F 2.5. Sections were stained with anti-CD123 by immunoperoxidase technique and counterstained with Mayer's hematoxylin. Magnifications: ×100 (left panels), ×500 (right panels). Two medium-power fields (corresponding to 0.3 mm^2^), selected on the basis of highest content of CD123+ positive cells, were evaluated and values were expressed as mean per mm^2^. Panels A, B and C respectively show tonsil sections from one representative healthy child, an HIV-infected child with abundant pDC and a HIV-infected child showing pDC depletion. In panel D are the number of pDC (CD123^+ ^cells) of tonsil specimens plotted against the CD4^+ ^cell percentage or the HIV RNA copy number of 8 HIV^+ ^children.

The decline of circulating pDC, correlating with reduction of CD4^+ ^and increased IFN-α-dependent MxA gene expression, in children with advanced HIV infection suggests that pDC might accumulate in secondary lymphatic tissues [39]. Thus we sought to determine if pDC (identified as CD123^+ ^cells) are present in lymph nodes and tonsils, previously obtained for diagnostic purposes from 8 HIV-infected children. The number of pDC identified in lymphoid tissues of HIV-infected children was extremely variable: in some specimens the pDC were abundant in the interfollicular areas of tonsils and lymph nodes of both age-matched control subjects (a section of tonsil tissue is shown as representative example in Figure 4A) and HIV^+ ^patients (a representative example is shown in Figure 4B), while in other HIV-infected children they were severely depleted (Figure 4C). It should be noted that pDC were regularly scant in cases showing general lymphoid depletion, but they are found to be rare also in cases with preserved lymphoid tissue, as indicated in Figure 4C. After plotting the number of pDC detected in lymphoid tissues of the HIV-infected children against the percentage of CD4^+ ^or against the HIV-RNA copy number, we observed a direct correlation between the number of lymphatic tissue-resident pDC and CD4^+ ^blood counts, while there was no correlation with HIV viral load (Figure 4D).

## Discussion

The relationship between pDC and CD4^+ ^cells is based on numerous concordant observations. Following HIV infection, both cell subsets decrease to levels associated with increased susceptibility to opportunistic infections [24]. Due to the fact that pDC infiltrate the medulla and the corticomedullary junction of the thymus, where they inhibit the replication of the virus [15], and due to the relationship between HIV infection and the release of newly generated T lymphocytes from the thymus into peripheral blood [26], we determined whether there was a direct relationship between the number of pDC and the extent of thymic output. We did not observe a correlation between the number of newly produced T lymphocytes containing TRECs and the number of pDC, most likely due to the fact that lymphocyte homeostasis is also regulated in peripheral lymphoid organs, depending on cell replication and removal [40].

Although higher pDC frequency found in long-term non-progressors [41] indicate that pDC also have a role in HIV infection control, a possible link also exists between disease outcome and reduced pDC number and function, including a decreased production of IFN-α by pDC during AIDS progression [24].

High plasma levels of IFN-α can be detected during acute HIV infection [42], but it is usually barely detectable in chronic HIV infection due to its short half-life or the rapid binding to its receptor [43-45]. Accordingly, we found low serum levels of IFN-α in HIV-infected children, but we detected significantly increased levels of IFN-α mRNA in blood cells, suggesting that pDC were in an active state and potentially capable to deliver the cytokine. The measurement of serum IFN-α and/or IFN-α mRNA might be used as markers of HIV disease progression and/or of therapy failure [42]. However, the biochemical and biological properties of the cytokine, including its limited distribution to blood circulation [17,44], present a serious limitation for its use in a clinical setting. On the contrary, the evaluation of MxA expression by real-time PCR might constitute a sensitive and reliable assay for measuring the extent of IFN-α biological activity. Since MxA mRNA is induced in response to IFN-α, the expression of MxA is strictly related to the amount of the cytokine [36]. Indeed, we have demonstrated that MxA mRNA expression increased above normal levels in ART-treated HIV-infected patients who had low numbers of both pDC and CD4^+ ^cells, suggesting that analysis of MxA induction could serve as a convenient marker for the evaluation of extent of pDC immune-restoration. In particular, MxA mRNA was distinctively increased in HIV-infected children with marked depletion of circulating pDC at levels that were proportional to viral load. This indicates that the failure to reach viral suppression in children with low CD4^+ ^and pDC leads to high increased levels of IFN-α. According to this hypothesis, we have observed that serum IFN-α concentration was significantly higher in HIV-infected children who have increased MxA mRNA values as compared to patients with MxA expression below the cut-off.

We have also observed that perinatally HIV-infected patients with low pDC number displayed the highest levels of MxA mRNA production. We speculate that MxA induction might be influenced by the interaction of IFN receptor with its other ligand, IFN-β, by an augmented IFN-α synthetic activity of fewer circulating pDC, or by the increased production of IFN-α by sources other than circulating pDC, such as human monocytes [46] or tissue-resident pDC. Indeed, since HIV infection up-regulates the pDC chemokine receptor CCR7 [47], these cells might accumulate in T-cell rich lymphoid tissues, such as tonsils or lymph nodes. Supporting this Dillon *et al*. [39] demonstrated an accumulation of partially activated pDC in lymphoid tissues. We did not observe a reduction of circulating pDC, but the immunohistochemistry results appear to argue against the hypothesis that enhanced tissue pDC migration is the main factor explaining the inverse correlation with MxA in perinatally HIV-infected patients. These issues will need further research, considering that Biancotto *et al*. [48] have demonstrated a reduction of pDC in nodes of chronically infected adults. Further, the number of pDC in lymphatic tissues of HIV^+ ^individuals might depend on the stage of the disease and on the extent of pDC activation and susceptibility to HIV-dependent cell death [39].

High levels of MxA have been reported in peripheral blood cells of patients with acute viral infections, including influenza, herpes, cytomegalovirus, rotavirus, adenovirus and RSV [36,49,50], but not in patients with HCV infection, therefore, this virus is considered a poor inducer of IFN-α [50,51]. Thus, MxA mRNA levels above the cut-off found in 3 out of 4 of our patients with HIV-HCV co-infection were probably correlated to the outcome of HIV infection.

## Conclusion

Although the interpretation of an elevated MxA level should be made on the basis of the complete clinical and laboratory data, our results indicate that the analysis of MxA may represent a valuable tool for the management of ART in perinatal HIV infection.

## Competing interests

The authors declare that they have no competing interests.
